# Growth of Ultrathin ZnCo_2_O_4_ Nanosheets on Reduced Graphene Oxide with Enhanced Lithium Storage Properties

**DOI:** 10.1002/advs.201400014

**Published:** 2015-01-21

**Authors:** Guoxin Gao, Hao Bin Wu, Bitao Dong, Shujiang Ding, Xiong Wen (David) Lou

**Affiliations:** ^1^ Department of Applied ChemistrySchool of ScienceXi'an Jiaotong UniversityXi'an710049P. R. China; ^2^ School of Chemical and Biomedical EngineeringNanyang Technological University62 Nanyang DriveSingapore637459

**Keywords:** nanosheets, graphene oxide, hybrid nanostructures, lithium storage, batteries, nanocomposites

## Abstract

The growth of ultrathin ZnCo_2_O_4_ nanosheets on reduced graphene oxide (denoted as rGO/ZnCo_2_O_4_) via a facile low‐temperature solution method combined with a subsequent annealing treatment is reported. With the assistance of citrate, interconnected ZnCo_2_O_4_ nanosheets can assemble into hierarchically porous overlays on both sides of rGO sheets. Such a hybrid nanostructure would effectively faciliate the charge transport and accommodate volume variation upon prolonged charge/discharge cycling for reversible lithium storage. As a result, the rGO/ZnCo_2_O_4_ nanocomposite manifests a very stable high reversible capacity of around 960 mAh g^−1^ over 100 cycles at a low current density of 90 mA g^−1^ and excellent rate capability.

## 1 Introduction

Lithium‐ion batteries (LIBs) have gained commercial success as the leading power source for portable electronics, and have shown great promise in upcoming large‐scale applications.^1^, ^2^, ^3^, ^4^ The ever‐growing market demands for LIBs have stimulated numerous research efforts aiming at the exploration of novel electrode materials with higher capacity and long‐term cycling stability.^5^, ^6^ Transi­tion metal oxides (TMOs), especially cobalt‐based oxides with a spinel structure, have been intensively investigated as potential alternatives to graphite‐based anode materials for their higher theoretical capacities.^7^, ^8^ However, cobalt‐based oxides are limited by their high cost and toxicity.^9^, ^10^ Thus, extensive research efforts are now made to fabricate novel ternary cobalt oxides by partially substituting Co with less expensive and eco‐friendly metals. Moreover, ternary metal oxides usually own many unique properties originated from the co‐existence of two types of different cations in a single crystal structure.^11^, ^12^, ^13^, ^14^, ^15^, ^16^, ^17^ Amongst a variety of candidates, ternary ZnCo_2_O_4_ has been considered attractive in view of its enhanced cycling stability and good environ­mental benignity.^16^, ^18^, ^19^ Importantly, ZnCo_2_O_4_ can store Li^+^ through not only the conversion reaction, but also the alloying/de‐alloying reaction between Zn and Li, which results in a high theoretical capacity of ca. 900 mAh g^−1^.^13^ Very recently, ZnCo_2_O_4_ anode materials with various morphologies such as nanoparticles, porous nano/microspheres and nanotubes/nanowires/nanorods have been synthesized and applied as anode materials for LIBs with high capacity.^4^, ^14^, ^16^, ^17^, ^18^, ^19^, ^20^, ^21^ Nevertheless, the intrinsic poor electric conductivity and low cycling stability due to drastic volume change during lithium insertion/extraction process still limit the practical application of ZnCo_2_O_4_‐based electrodes.^17^

To circumvent these problems, one effective strategy is to employ a suitable flexible matrix to accommodate the volume variation and improve the electric conductivity at the same time.^22^ In this regard, graphene or reduced graphene oxide (rGO), has been widely investigated and proven as an effective conducting support to host TMOs in high‐power LIBs, because of its outstanding characteristics, including high electrical conductivity, excellent mechanical flexibility, large specific surface area, and high chemical stability.^23^, ^24^, ^25^, ^26^, ^27^, ^28^, ^29^ Generally, rGO in the nanocomposites could not only increase the electric conductivity, but also provide an elastic buffering support to withstand the huge volume change and drastic structural re‐organization of TMOs, thus leading to improved cycling stability.^25^, ^26^ Although tremendous efforts have been devoted to coupling graphene with different TMOs, hybrid nanostructures of graphene supported ZnCo_2_O­_4_ nanosheets as electrode materials for LIBs have not been realized so far.

Herein, we develop a facile two‐step strategy to design and fabricate a unique hierarchical hybrid structure of rGO supported ZnCo_2_O_4_ nanosheets (denoted as rGO/ZnCo_2_O_4_) as an advanced anode material for high performance LIBs. With the assistance of trisodium citrate, ultrathin ZnCo_2_O_4_ nanosheets can assemble into a hierarchically porous film that fully covers both sides of rGO sheets. With the structural and compositional advantages, the as‐synthesized rGO/ZnCo_2_O_4_ nanocomposite is expected to manifest enhanced lithium storage properties.

## 2 Experimental Section

### 2.1 Materials Synthesis

Graphene oxide (GO) was first synthesized based on a modified Hummer's method.^30^ In a typical synthesis of reduced GO (rGO) supported ZnCo_2_O_4_ nanosheets hybrid nanostructure, 10 mg of GO was first dispersed in 40 mL of deionized (DI) water by ultrasonication for 30 min. Then 0.2 mmol of Zn(NO_3_)_2_.6H_2_O, 0.4 mmol of Co(NO_3_)_2_.6H_2_O, 0.5 mmol of hexamethylenetetramine (HMT) and 0.15 mmol of trisodium citrate dihydrate (TSC) were added into the above solution. After ultrasonication for another 5 min, the mixed solution was refluxed in an oil bath at 90 °C for 6 h. After being collected by centrifugation and rinsed with DI water and ethanol for several times, the obtained Zn‐Co precursor grown on rGO was dried overnight at 80 °C. Finally, the product was annealed at 400 °C for 3 h in N_2_ atmosphere with a slow heating rate of 1 °C min^−1^ to generate well‐defined rGO‐supported ZnCo_2_O_4_ nanosheets.

### 2.2 Materials Characterization

X‐ray diffraction (XRD) patterns were obtained on a Bruker D2 Phaser X‐Ray Diffractometer with Ni filtered Cu Kα radiation (λ = 1.5406 Å) at a voltage of 30 kV and a current of 10 mA. Field‐emission scanning electron microscope (FESEM) images were obtained by a JEOL JSM‐6700F microscope operated at 5 kV. Transmission electron microscope (TEM) images were recorded by JEOL JEM‐2010 and JEOL JEM‐2100F microscopes. Thermogravimetric analysis (TGA) was carried out under air flow of 200 mL min^−1^ with a temperature ramp of 10 °C min^−1^. Nitrogen sorption measurement was acquired on Autosorb 6B at –196 °C.

### 2.3 Electrochemical Measurements

The electrochemical tests were conducted in two‐electrode Swagelok cells. The working electrodes consisted of 70 wt% of active materials, 20 wt% of conductive carbon black (Super‐P‐Li), and 10 wt% of polymer binder (polyvinylidene fluoride, PVDF). The electrolyte is 1 M LiPF_6_ in a mixture of ethyl­ene carbonate and diethyl carbonate (1:1 by weight). Lithium discs were used as both the counter electrode and reference electrode. Cell assembly was carried out in an Ar‐filled glovebox (Inno­vative Technology Inc.) with moisture and oxygen concentrations below 1.0 ppm. The galvanostatic charge‐discharge measurements were performed within a voltage window of 0.01–3 V on a NEWARE battery tester.

## 3 Results and Discussion

**Figure 1:**
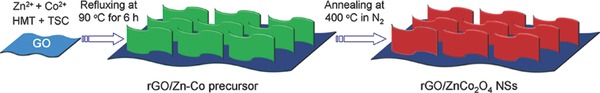
Schematic illustration of the formation of rGO/ZnCo_2_O_4_ hybrid structure.

In the present synthesis, two steps are involved to synthesize hierarchical ZnCo_2_O_4_‐rGO, as illustrated in **Figure** **1**. Specifically, GO sheets are first dispersed into an aqueous solution containing Zn(NO_3_)_2_, Co(NO_3_)_2_, hexamethylenetetramine (HMT) and trisodium citrate (TSC). During the refluxing process, decomposition of HMT results in the formation of Zn‐Co precursor. Due to the strong coordination effect between the function groups of GO sheets and metal ions, the Zn‐Co precursor selectively grows on the surface of GO sheets. Besides, it's worth mentioning that the hydrolysis of TSC can further promote formation of Zn‐Co precursor into unique ultrathin nanosheets standing upright on both sides of the GO sheets (denoted as rGO/Zn‐Co precursor). In the growth process, GO is expected to be partially reduced by the reducing species generated from HMT and TSC. In the second step, the Zn‐Co precursor can be easily transformed to crystalline ZnCo_2_O_4_ with well‐retained nanosheets morphology via a facile thermal annealing treatment in N_2_ at 400 °C. As a result, the novel rGO/ZnCo_2_O_4_ hierarchical hybrid structure can be obtained.

**Figure 2:**
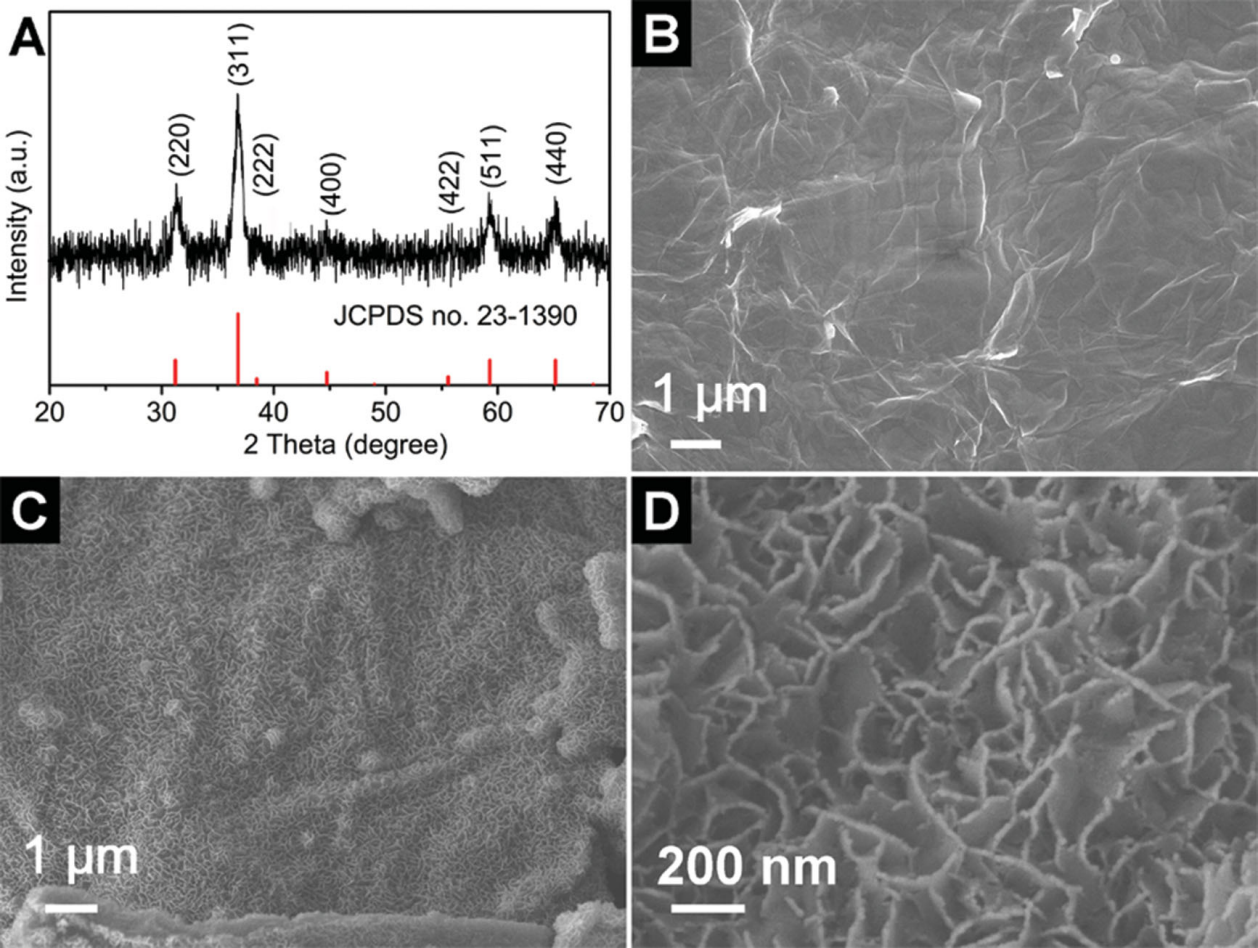
A) XRD pattern of the rGO/ZnCo_2_O_4_ nanocomposite. FESEM images of B) GO; and C,D) rGO/ZnCo_2_O_4_ hybrid structure obtained with 0.15 mmol of TSC added.

The obtained nanocomposite is first characterized by powder X‐ray diffraction (XRD) to determine their crystallographic structures. The as‐prepared Zn‐Co precursor is nearly amorphous (Figure S1, see Supporting Information). However, after annealing at 400 °C in N_2_ for 3 h, all of the identified diffraction peaks in the XRD pattern of the annealed product confirm the formation of the spinel ZnCo_2_O_4_ phase (JCPDS card no. 23–1390) without noticeable signals of possible crystalline impurities (**Figure** **2**A).^13^, ^17^ The morphology and structure of the pristine GO sheets and as‐prepared rGO/ZnCo_2_O_4_ hybrid are further examined by field‐emission scanning electron micro­scopy (FESEM). Figure 2B shows that the surface of the pristine GO sheets is very clean and flat. After the solution reaction and post‐annealing treatment in N_2_, crystalline ZnCo_2_O_4_ nanosheets are grown on the reduced GO sheets. As shown in Figure 2C, nanosheets uniformly cover the whole surface of rGO to form a coating layer, demonstrating the strong coupling effect between ZnCo_2_O_4_ nanosheets and the rGO surface. Better revealed by the high‐magnification FESEM image in Figure 2D, ultrathin ZnCo_2_O_4_ nanosheets on rGO sheets can form a well‐developed three‐dimensional interconnected porous network standing upright on the flexible rGO sheets. However, without the support of GO sheets, it is found that only flower‐like ZnCo_2_O_4_ microspheres composed of nanosheets are obtained under the same conditions (Figure S2, see Supporting Information), suggesting that the GO substrate is able to prevent the severe aggregation of ZnCo_2_O_4_ nanosheets effectively. The highly porous feature of the composite is characterized by N_2_ adsorption‐desorption measurement (Figure S3, see Supporting Information), which reveals a high Brunauer‐Emmett‐Teller (BET) specific surface area of about 186.6 m^2^ g^−1^. In addition, thermogravimetric analysis (TGA) shows that the weight fraction of the rGO support is about 13.7 wt% in the final nanocomposite (Figure S4, see Supporting Information). Such a porous architecture with conductive graphene support holds great promise in offering sufficient surface area to facilitate electrochemical reactions thus delivering excellent electrochemical performance.

**Figure 3:**
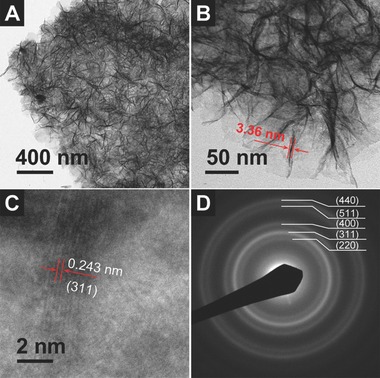
A,B) TEM, C) HRTEM images and D) SAED pattern of the rGO/ZnCo_2_O_4_ obtained with 0.15 mmol of TSC added.

The intriguing structure is also elucidated under transmission electron microscopy (TEM) to provide further insight about the morphology and structure of the as‐prepared rGO/ZnCo_2_O_4_ hierarchical hybrid. In good agreement with the FESEM results, a low‐magnification TEM image (**Figure** **3**A) shows that numerous ultrathin ZnCo_2_O_4_ nanosheets are quite loosely packed on the well‐retained micro‐sized rGO sheets. With a closer observation, the nanosheets are around 3.4 nm in thickness (Figure 3B). Due to the low contrast of graphene and the presence of large amount of ZnCo_2_O_4_ nanosheets, the graphene support cannot be directly observed under TEM. In addition, the porous structure of these ultrathin ZnCo_2_O_4_ nanosheets can be clearly observed in a high‐magnification TEM image (Figure S5, see Supporting Information), which agrees well with the above BET analysis. The formation of porous structure is mainly due to the gradual decomposition of the precursor (hydroxide and carbonate) during the annealing process.^24^ Consistent with XRD analysis, a set of distinct lattice fringes with a spacing of 0.24 nm can be observed in the high‐resolution TEM image of a typical ZnCo_2_O_4_ nanosheet (Figure 3C), which corresponds to the (311) crystal planes of the spinel ZnCo_2_O_4_ phase.^31^ Furthermore, the selected area electron diffraction (SAED) pattern (Figure 3D) indicates a polycrystalline nature of the nanosheets and the diffraction rings can be readily assigned to the crystal planes of the spinel ZnCo_2_O_4_ phase.

**Figure 4:**
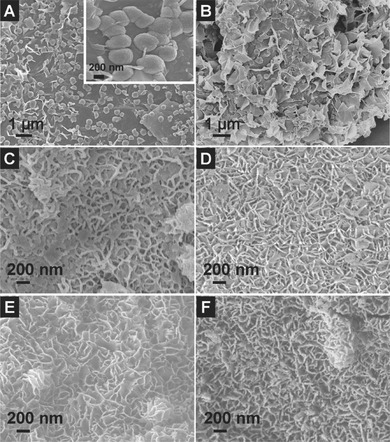
FESEM images of rGO/ZnCo_2_O_4_ obtained with various amounts of TSC: A) 0 mmol, B) 0.025 mmol, C) 0.05 mmol, D) 0.10 mmol, E) 0.15 mmol, and F) 0.25 mmol.

It is worth mentioning that the presence of TSC in the reaction plays a crucial role in the formation of the hierarchical hybrid structure of ultrathin nanosheet subunits on the rGO substrate.^28^, ^32^ Without the addition of TSC, only some irregular ZnCo_2_O_4_ nanoparticles or nanospheres can be found on the surface of rGO substrate (**Figure** **4**A). When a small amount of TSC (0.025 mmol) is added, most ZnCo_2_O_4_ nanoparticles have evolved into large and irregular nanosheets lying on the surface of rGO (Figure 4B), which indicates that heterogeneous nucleation of Zn‐Co precursor nanosheets on GO support has been facilitated by the functional groups of TSC. Increasing the amount of TSC to 0.05 mmol, these irregular nanosheets evolve into slender nanosheets, which start to stand on the surface of rGO substrate (Figure 4C). Further increasing the amount of TSC leads to the formation of large and up‐standing nanosheets on GO sheets with high uniformity. In particular, when the amount of TSC is 0.15 mmol, the as‐prepared hybrid structure manifests the optimal morphology, which consists of densely standing and interconnected nanosheets (Figure 4E). Nevertheless, upon increasing the amount of TSC to 0.25 mmol, the packing of nanosheets becomes denser and some agglomeration starts to appear on the surface of rGO sheets (Figure 4F). Clearly, the morphology of the hierarchical rGO/ZnCo_2_O_4_ hybrid structure can be tuned by simply controlling the amount of TSC in the reaction solution through the possible coordination effect between metal ions and functional groups of TSC. Meanwhile, HMT simply serves as the alkaline source to trigger the formation of Zn‐Co precursor with sphere‐like nanostructures firmly anchoring onto rGO sheets.^33^ Without TSC, HMT can only lead to the precipitation of irregular particles of Zn‐Co precursor on rGO sheets (Figure S6, see Supporting Information).

**Figure 5:**
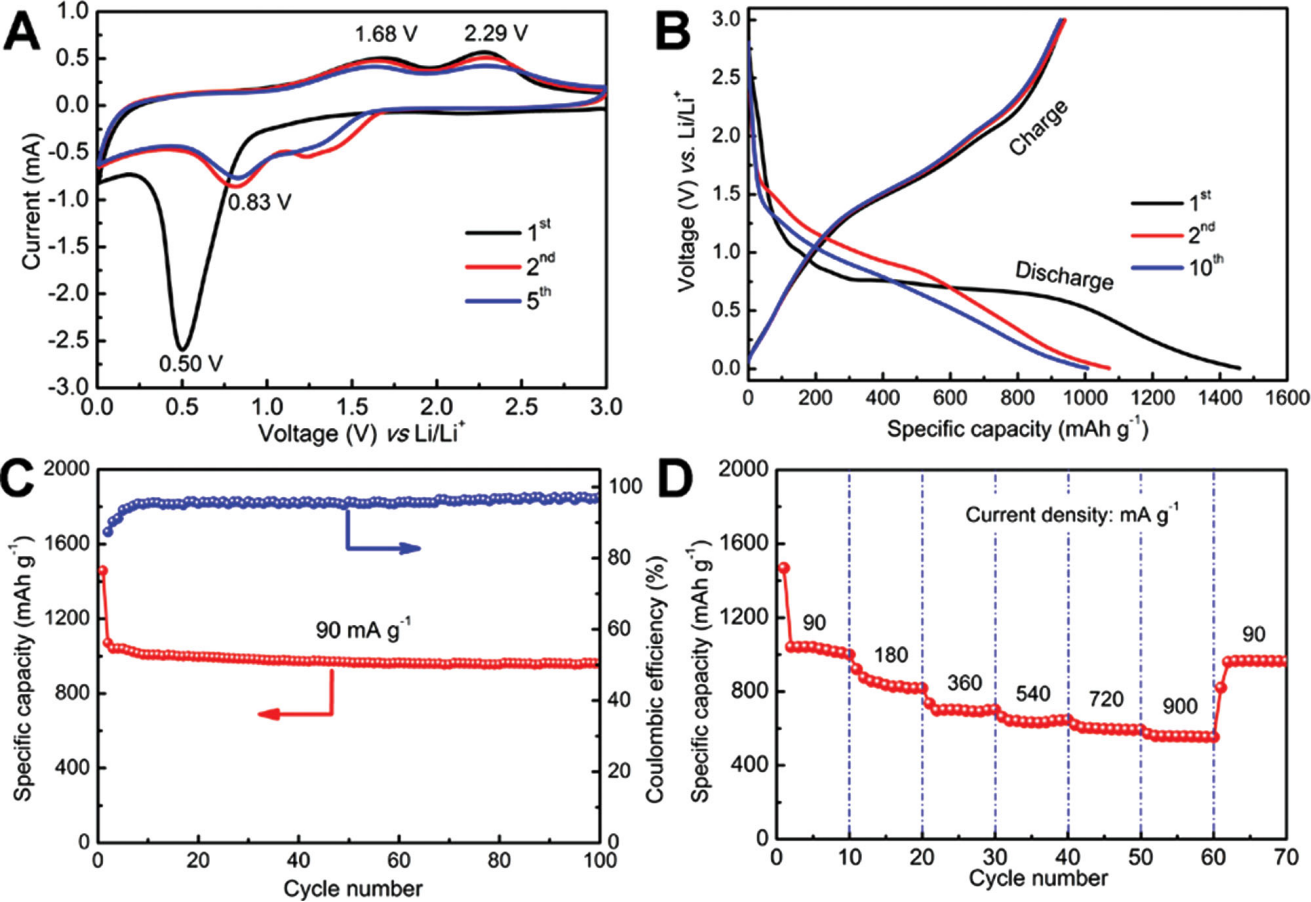
Electrochemical characterization of the rGO/ZnCo_2_O_4_ nanocomposite: A) CVs at a scan rate of 0.5 mV s^−1^; B) discharge–charge voltage profiles; C) cycling performance at a current density of 90 mA g^−1^ and corresponding Coulombic efficiency, and D) rate capability at different current densities. All measurements were conducted over the voltage range of 0.01–3.0 V vs. Li/Li^+^.

We next evaluate electrochemical properties of the rGO/ZnCo_2_O_4_ nanocomposite as an anode material for LIBs. **Figure** **5**A shows representative cyclic voltammograms (CVs) for the 1^st^, 2^nd^ and 5^th^ cycles at a scan rate of 0.5 mV s^−1^ in the voltage window of 0.01–3.0 V vs. Li/Li^+^. Consistent with previous studies of ZnCo_2_O_4_ anodes, several redox current peaks can be clearly identified from the CVs, indicating the similar electrochemical reaction mechanism.^13^, ^18^ In the first cycle, the irreversible cathodic peak located at around 0.50 V can be attributed to the reduction of ZnCo_2_O_4_ to metallic Zn and Co. The significant decrease in the peak intensity in the subsequent scans indicates the existence of some irreversible processes during the first cycle, whereas the shift of peak position to higher potentials in the following cycles might be related to some activation process for the Li^+^ insertion in the first cycle. Meanwhile, two broad anodic peaks centered at about 1.68 and 2.29 V in the following anodic scan can be ascribed to the oxidation of metallic Zn and Co to ZnO and CoO_x,_ respectively. Thus, on the basis of above CV analysis and previous reported lithium storage mechanisms of ZnO, CoO and Co_3_O_4_, the lithium insertion/extraction reactions for our rGO/ZnCo_2_O_4_ electrode might be described as follows:^19^, ^34^

(1) $${\mathrm{ZnCo}}_{2}O_{4}\;+\;8\;{\mathrm{Li}}^{+}\;+\;{\mathrm{8e}}^{-}\;\to \;\mathrm{Zn}\;+\;\mathrm{2Co}\;+\;4\;{\mathrm{Li}}_{2}O$$

(2) $$\mathrm{Zn}\;+\;{\mathrm{Li}}^{+}\;+\;e^{-}\;↔\;\mathrm{LiZn}$$

(3) $$\mathrm{Zn}\;+\;{\mathrm{Li}}_{2}O\;↔\;\mathrm{ZnO}\;+\;2\;{\mathrm{Li}}^{+}\;+\;{\mathrm{2e}}^{-}$$

(4) $$2\;\mathrm{Co}\;+\;2\;{\mathrm{Li}}_{2}O\;↔\;\mathrm{2CoO}\;+\;4\;{\mathrm{Li}}^{+}\;+\;{\mathrm{4e}}^{-}$$

(5) $$2\;\mathrm{CoO}\;+\;{\mathrm{2/3Li}}_{2}O\;↔\;\mathrm{2/3}\;{\mathrm{Co}}_{3}O_{4}\;+\;\mathrm{4/3}\;{\mathrm{Li}}^{+}\;+\;\mathrm{4/3}\;e^{-}$$

Typical discharge‐charge voltage profiles of rGO/ZnCo_2_O_4_ at a current density of 90 mA g^−1^ are shown in Figure 5B. The hybrid rGO/ZnCo_2_O_4_ anode material delivers high first‐cycle discharge and charge capacities of 1457.8 and 936.3 mAh g^−1^, respectively. The corresponding irreversible loss of about 36% during the first charge is commonly attributed to irreversible side reactions on the surface of electrodes, such as formation of solid‐electrolyte interface (SEI) layer, and possible incomplete restoration of metallic Zn and Co into the original oxides.^13^, ^16^, ^34^, ^35^, ^36^ Nevertheless, the voltage profiles approximately overlap except for the initial discharge, indicating good reversibility of the electrochemical reactions of the material for reversible lithium storage. To evaluate the cycling stability, the rGO/ZnCo_2_O_4_ electrode is charged and discharged at a current density of 90 mA g^−1^, as depicted in Figure 5C. As expected, the rGO/ZnCo_2_O_4_ nanocomposite shows good capacity retention from the second cycle onwards and eventually delivers a reversible discharge capacity as high as 960.8 mAh g^−1^ in the 100^th^ cycle, corresponding to 89.7% of the second‐cycle discharge capacity. As a comparison, without the rGO sheets support, the electrode of flower‐like ZnCo_2_O_4_ microspheres shows poor cycling stability (Figure S7, see Supporting Information). After 100 cycles at the same current density, its discharge capacity decreases sharply to 421.6 mAh g^−1^, corresponding to about 43% of the second‐cycle discharge capacity. Owing to the unique structure, the rGO/ZnCo_2_O_4_ nanocomposite manifests excellent capacity retention at continuously varying current densities ranging from 90 to 900 mA g^−1^ as shown in Figure 5D. The specific capacity of rGO/ZnCo_2_O_4_ decreases steadily as the current density increases, but still retains high values. For example, at a high current density of 900 mA g^−1^, the rGO/ZnCo_2_O_4_ electrode is still able to deliver a stable discharge capacity of about 593.2 mAh g^−1^. Remarkably, the capacity could resume to a high value of 963.7 mAh g^−1^ when the current density is reduced back to 90 mA g^−1^, indicating good reversibility of the electrode material. Moreover, because of its excellent robustness, the morphology and structure of the rGO/ZnCo_2_O_4_ nanocomposite are perfectly retained after ten charge‐discharge cycles at 90 mA g^−1^ (Figure S8, see Supporting Information).

Clearly, the rationally designed nanostructure and composition of rGO/ZnCo_2_O_4_ are beneficial for the enhanced electrochemical performance. Specifically, the hierarchically porous structure assembled by ultrathin ZnCo_2_O_4_ nanosheets on the flexible rGO substrate provides sufficient electrode‐electrolyte contact area for high Li^+^ ion flux across the interface and at the same time shortens Li^+^ ion diffusion distance, thus greatly facilitating the electrochemical processes especially at high current densities.^37^ On the other hand, open and porous frameworks and the flexible rGO substrate could better improve the electrode stability by effectively mitigating the internal mechanical stress during repeated charging‐discharging processes, as well as preventing the nanostructures from agglomeration.^38^ Finally, the rGO substrate with relatively good electrical conductivity might improve the reaction kinetics towards fast lithium insertion/extraction.^9^, ^39^

## 4 Conclusion

In summary, we have developed a simple strategy to grow ultrathin ZnCo_2_O_4_ nanosheets onto reduced graphene oxide (rGO) sheets for enhanced lithium storage properties. The synthesis involves growth of precursor nanosheets on rGO surface and a subsequent thermal treatment. The morphology of this novel hybrid structure can be controlled by the added amount of trisodium citrate (TSC) in the reaction solution. The hierarchical rGO/ZnCo_2_O_4_ nanocomposite demonstrates high reversible lithium storage capacity of 960.8 mAh g^−1^ over 100 cycles at the current density of 90 mA g^−1^, and remarkable capacity retention at increased current densities as an advanced anode material for LIBs. Therefore, the present work offers a simple and effective approach for the development of high‐performance electrode materials for advanced lithium ion batteries.
